# Antecedent soil moisture prior to freezing can affect quantity, composition and stability of soil dissolved organic matter during thaw

**DOI:** 10.1038/s41598-017-06563-8

**Published:** 2017-07-25

**Authors:** Haohao Wu, Xingkai Xu, Weiguo Cheng, Pingqing Fu, Fayun Li

**Affiliations:** 1 State Key Laboratory of Atmospheric Boundary Layer Physics and Atmospheric Chemistry, Institute of Atmospheric Physics, Chinese Academy of Sciences, Beijing, 100029 China; 2 Department of Atmospheric Chemistry and Environmental Science, College of Earth Science, University of Chinese Academy of Sciences, Beijing, 100049 China; 3 Faculty of Agriculture, Yamagata University, Tsuruoka, 997-8555 Japan; 4 Institute of Eco-Environmental Sciences, Liaoning Shihua University, Fushun, 113001 China; 5 National & Local United Engineering Laboratory of Petroleum Chemical Process Operation, Optimization and Energy Conservation Technology, Liaoning Shihua University, Fushun, 113001 China

## Abstract

There are large amounts of dissolved organic matter (DOM) released into the soil during spring thaw, but its bioavailability and components are still unknown. The quantity, composition and stability of DOM in water extracts of forest soils during thaw were studied after two-month freezing with 9 levels of soil moisture ranging from 10% to 90% water-filled pore space (WFPS), by measuring soil carbon dioxide (CO_2_) flux, biodegradable dissolved organic carbon (BDOC) and nitrogen (BDON), ultraviolet absorbance and parallel factor analysis of fluorescence excitation-emission matrices. Concentrations of BDOC, BDON, DOC and DON were lowest around 30% WFPS and relatively higher and lower soil moisture both increased DOM and BDOM concentrations in thawing soil. With increasing WFPS, the dominant component of soil DOM changed from humic acid-like substances to fulvic acid-like substances and the biological origin of DOM increased gradually. The protein-like component accounted for 8–20% of soil DOM and was affected by vegetation type and WFPS singly and interactively. The results implied that forest soils with more than 50% WFPS before winter freezing could release large amounts of fulvic acid-like DOM, which would be easily biodegraded and emitted as CO_2_ or run off with ground water during spring snow thaw.

## Introduction

Future climatic change is likely to increase the occurrence of soil freezing-thawing events in high latitude and/or high altitude zones^1^. Deep and long-term freezing during winter causes physical damages to soil aggregates and brings about the lysis of soil organisms, which can lead to a release of dissolved organic matter (DOM) into the soil during spring thaw^2–4^. The DOM would be absorbed in the soil, run off and exported into adjacent aquatic habitats with melted snow or discharged into atmosphere as greenhouse gases through microbial functions^5–8^. For example, *Rember and Trefry* ^9^ reported that a large pulse of dissolved organic carbon (DOC) incorporated with snowmelt from thawing soil layers led to an increase of DOC concentration by 2 to 6 times in adjacent rivers. Dissolved organic nitrogen (DON) released into the soil during thaw is a substrate for N mineralization and the subsequent nitrous oxide (N_2_O) production including nitrification and denitrification. Increased N mineralization and fluxes of carbon dioxide (CO_2_) and N_2_O during soil thawing has been observed in various terrestrial ecosystems^3, 4, 8^. Ultimately, the fate of soil DOM released during thaw can depend on its quality, such as aromaticity, mobility and degradation^10^. Unfortunately, recent studies have focused mainly on the quantity of DOM released during thaw rather than its components and biological decomposition. Thus, determining both the quantity and quality of DOM released into the soil after freezing would improve our understanding of its environmental influence during spring thaw.

At the onset of winter freezing, soil moisture varied greatly due to the changes in precipitation pattern, vegetation coverage and soil permeability^11–13^. High antecedent soil moisture prior to freezing results in more ice, which can increase physical damages to soil aggregates and microorganisms and then further causes soil DOM release^4, 14^. The biological use of this DOM pulse finally led to a spike in CO_2_ flux during thaw^4, 15^. However, *Teepe et al*.^16^ reported that the CO_2_ flux decreased with increasing soil moisture ranging from 42% to 76% water-filled pore space (WFPS) during freezing-thawing cycle periods. The inconsistence is partly related to changes in the quantity and quality of DOM released into the soil during thawing, because it serves as a potential nutrient source to soil microorganisms and controls the growth efficiency of heterotrophic bacteria^17–19^. Furthermore, the influence of soil moisture prior to freezing on the components and biodegradation of DOM released during thaw is still unclear so far. As far as we know, only *Haei et al*.^10^ reported the changes in the aromaticity of DOM, which released into the soil after freezing at 30%, 60% and 90% WFPS, using carbon-specific ultraviolet absorbance at 254 nm (SUVA_254_). However, the SUVA_254_, which indicates the aromaticity of DOM, is insufficient for understanding the complex components of DOM released during thaw^20, 21^. It is noteworthy fluorescence excitation–emission (EEM) spectrophotometry combined with parallel factor (PARAFAC) analysis has been shown to be a sensitive, rapid and non-destructive technique to provide quantitative information on the components of DOM (e.g. humic-like, fulvic-like and protein-like DOM) from terrestrial and aquatic sources^22–25^, and biodegradability of DOM can be measured by soil extracts plus inoculum incubation experiment^26, 27^. Combined with the environmental impacts (e.g. greenhouse gases fluxes and nutrient leaching) caused by the soil DOM released during spring thaw, it is important to understand the influence of antecedent soil moisture prior to freezing on the quantity, composition and stability of soil DOM during thaw, by using EEM spectrophotometry with PARAFAC and the DOM degradation incubation experiment.

The seasonally or perennially frozen soils of boreal forests contain one of the largest pools of carbon in the terrestrial biosphere^28^. Broadleaf and Korean pine mixed forest (BKPF) is the major component of forest ecosystems in Changbai Mountains in northeastern China. In such area, the mature mixed forest lies in climax community of forest succession, with a greater soil organic matter content and lower bulk density than an adjacent secondary white birch forest (WBF) (Supplementary Table S1). Due to relatively lower vegetation coverage and phototaxis property, soil available nutrients, microbial properties and hydrothermal conditions under the white birch forest stand are different from those under the mature mixed forest. The differences in soil hydrothermal conditions, chemical and microbial properties under the two forest stands can affect the concentrations, components and stability of DOM released into the soil during spring thaw. Increasing antecedent soil moisture prior to freezing can promote the DOM released into the soil and affects soil CO_2_ flux during thawing, thus we hypothesize an accompanying variation in the components and biodegradation of the released DOM. To study the effect of soil moisture on the quantity and quality of DOM released into the thawing soil, a simulated freezing-thawing experiment was done using soils sampled from the two forest stands. The concentrations, components and optical properties of DOM released into the soil during thawing were measured by using EEM spectrophotometry with PARAFAC analysis and ultraviolet absorbance. Together with CO_2_ flux from thawing soil and the biodegradation properties of DOM, this study would provide quantitative information on the concentrations, compositions and stability of DOM released into the thawing soils after freezing with 9 levels of soil moisture ranging from 10% to 90% WFPS. The results would be beneficial for our understanding of the properties and environmental functions of DOM released into the soil during spring thaw in cold temperate zones.

## Results

### Concentrations and biodegradation of DOM

**Figure 1:**
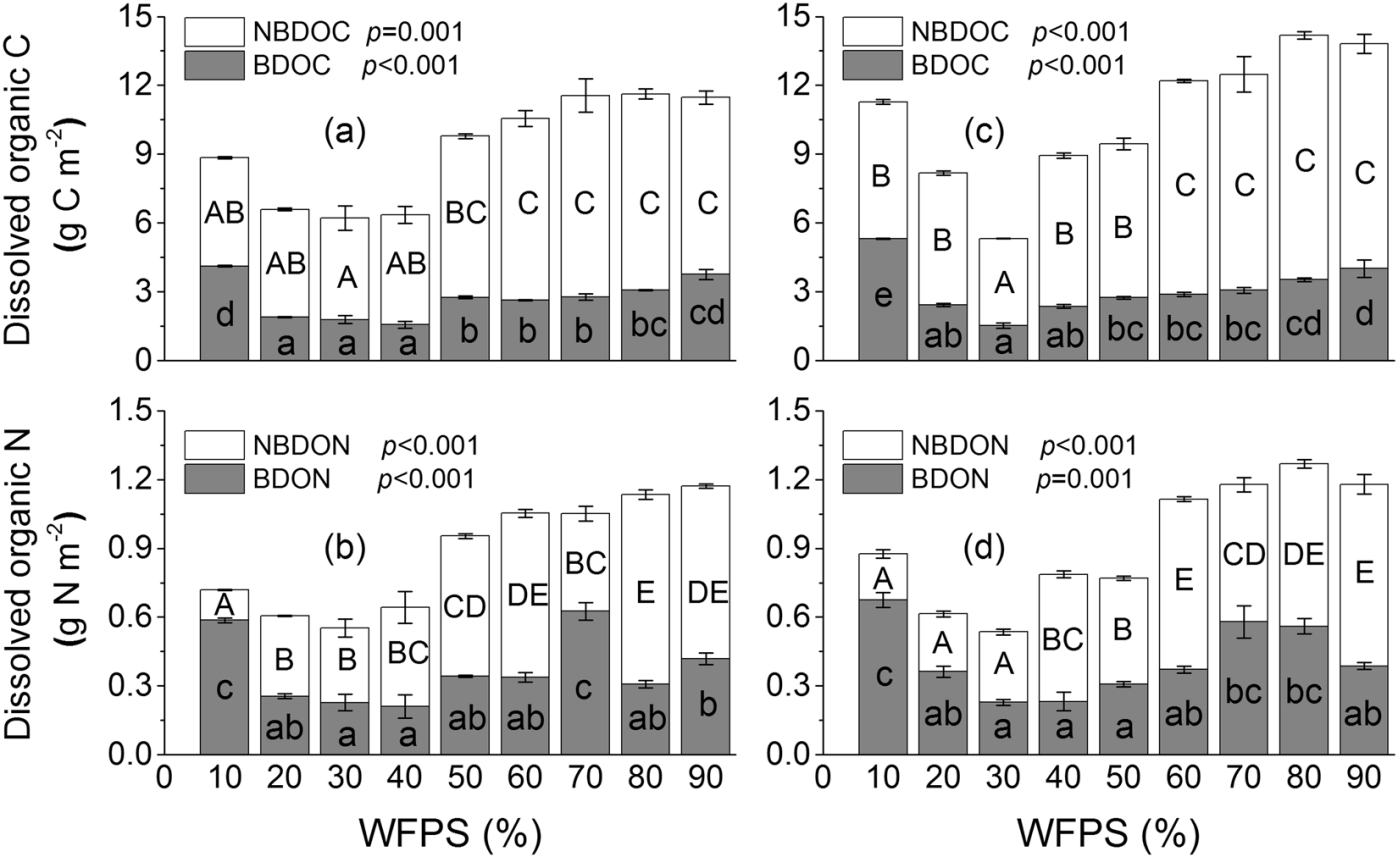
Effects of soil moisture on BDOM and NBDOM concentrations of soil water extracts. (**a**) and (**b**) represent for WBF soil; (**c**) and (**d**) represent for BKPF soil. Different lowercase and capital letters indicate significant differences of BDOM and NBDOM concentrations between different soil moisture levels at the *p* < 0.05 level, respectively. Significant levels for the effect of soil moisture on BDOM and NBDOM pools are shown inside each sub-plot. Error bars reflect the standard error of BDOM and NBDOM concentrations between replicates, respectively (*n* = 3 for all treatments).

The variation of WFPS significantly affected the concentrations of biodegradable DOC (BDOC), non-biodegradable DOC (NBDOC), biodegradable DON (BDON) and non-biodegradable DON (NBDON) in water extracts of WBF and BKPF soils during thaw (*p* ≤ 0.001) (Fig. 1). Concentrations of BDOC, NBDOC and DOC were generally smallest at around 30% WFPS in the two forest soils, especially in the BKPF soil (Fig. 1a,c). The concentration of BDOC was largest at 10% WFPS in the two forest soils, especially in the BKPF soil (*p* < 0.05), and it generally increased from 30% to 90% WFPS in the two forest soils (Fig. 1a,c). Compared to the 30% WFPS treatment, a larger concentration of NBDOC in the two forest soils generally occurred at high soil moisture ranging from 50% to 90% WFPS (*p* < 0.05) (Fig. 1a,c). The concentration of BDON in the WBF soil was significantly larger at 10% and 70% WFPS than that at the other soil moisture levels (*p* < 0.05) (Fig. 1b). For water extracts of BKPF soil, BDON concentration was largest at 10% WFPS, which was not significantly different from that at 70% and 80% WFPS (Fig. 1d). Similar to the NBDOC, the concentration of NBDON generally remained to be larger at high soil moisture ranging from 50% to 90% WFPS, expect for the 70% WFPS treatment of WBF soil (Fig. 1b,d).

### Features of EEM and components of DOM by PARAFAC analysis

**Figure 2:**
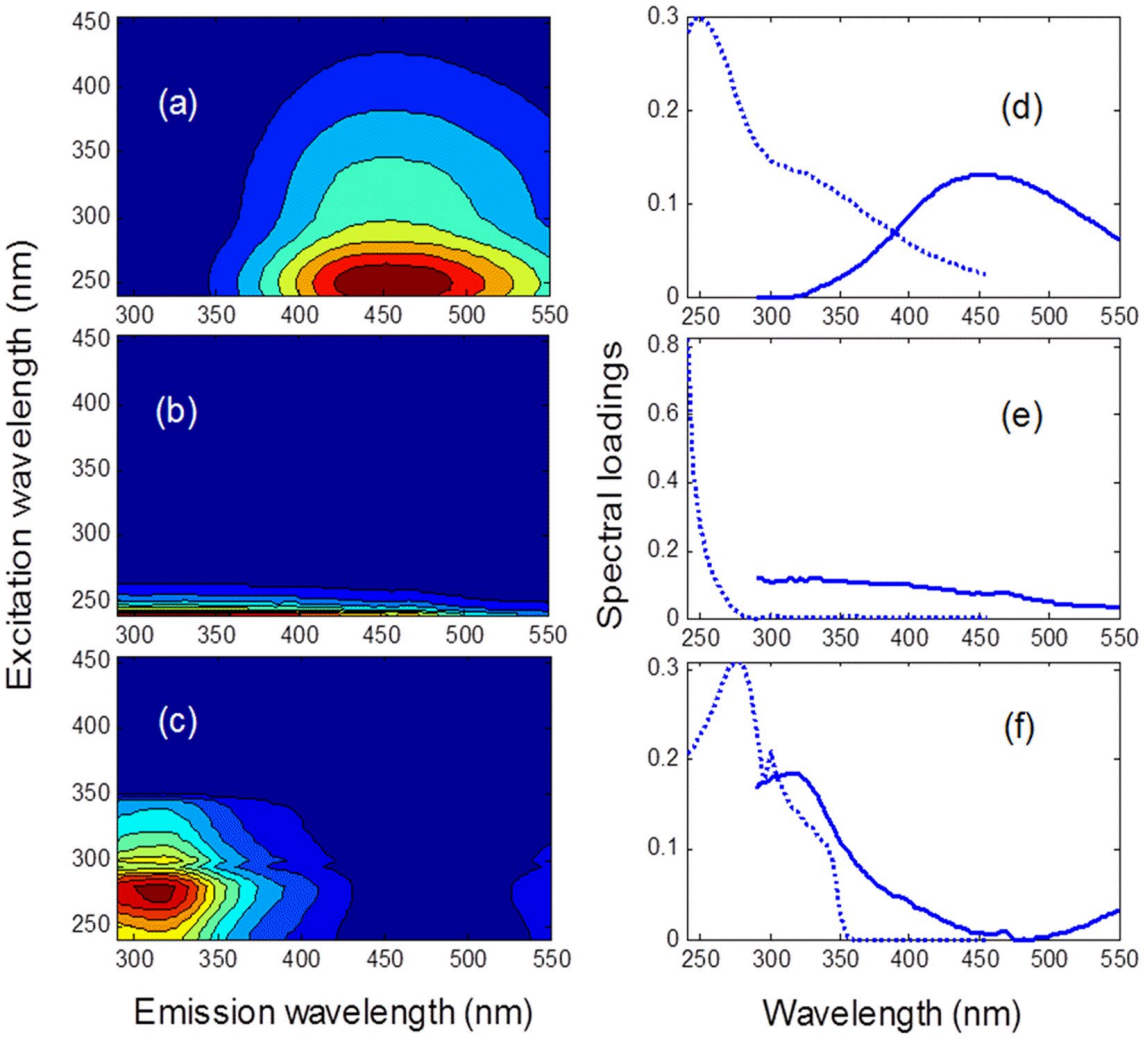
EEMs and spectral loadings of three components modeled by PARAFAC in water extracts of thawing forest soils. (**a**) and (**d**) represent for component 1; (**b**) and (**e**) represent for component 2; (**c**) and (**f**) represent for component 3. The dotted lines show the excitation loadings, and the solid lines represent the emission loadings.

Typical EEM fluorescence spectra of water extract of BKPF soil at 10% WFPS during thaw was displayed in Supplementary Figure S1. There were three fluorescence peaks, which represented humic acid-like component, fulvic acid-like component and protein-like component, respectively. After PARAFAC analysis of all the 54 EEMs of water extracts, the three components were validated though split half analysis and explained 99.45% of the variation in the data. Peak of component 1 occurred at the location where excitation wavelength (λ_Ex_) was around 250 nm and emission wavelength (λ_Em_) ranged from 440 nm to 460 nm (Fig. 2a,d). Peak of component 2 located at the area where λ_Ex_ was less than 250 nm (Fig. 2b,e). Peak of component 3 contained two peaks: the dominant one was observed at λ_Ex_/λ_Em_ = 270–290 nm/300–340 nm and the other one was observed at λ_Ex_/λ_Em_ = 300 nm/300–340 nm (Fig. 2c,f).

**Figure 3:**
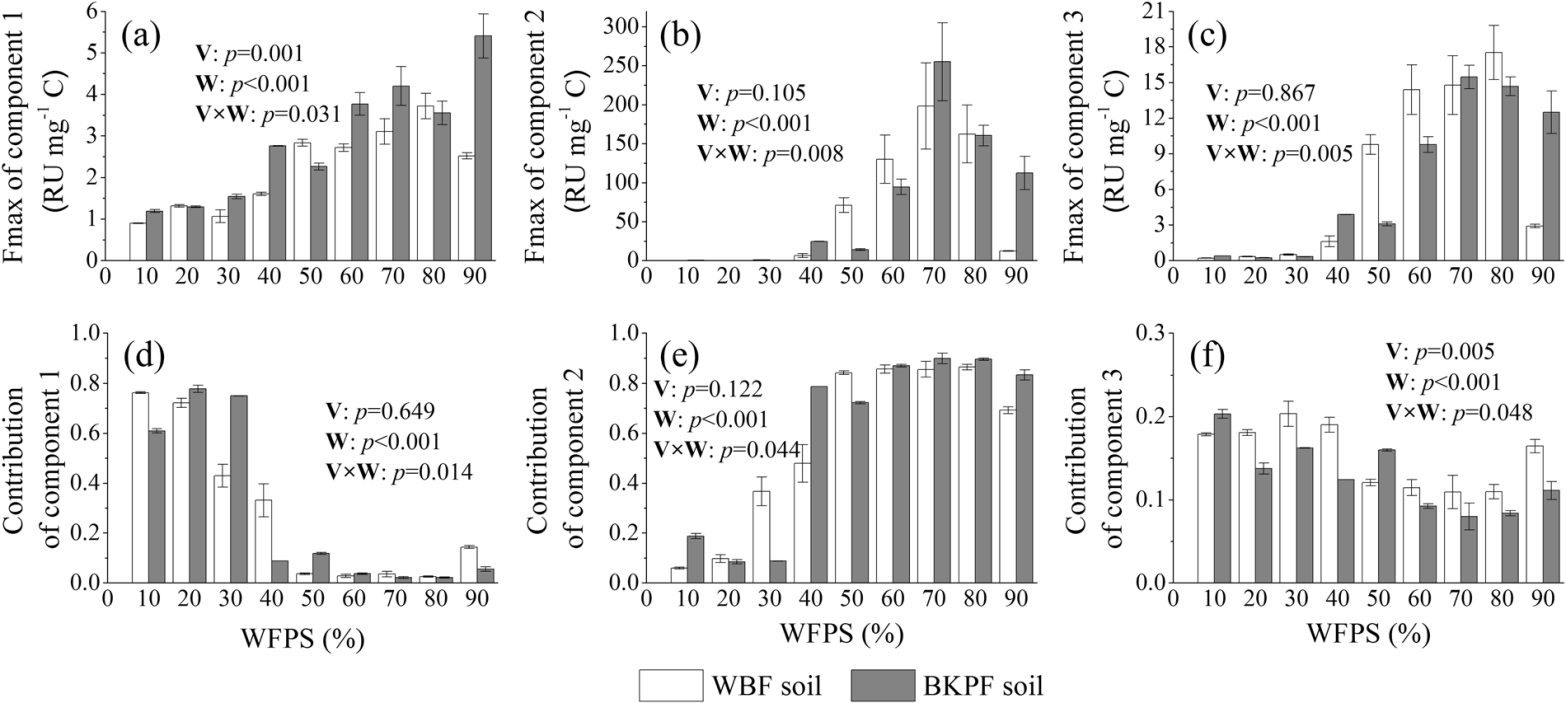
Effects of soil moisture on Fmax and contribution of three components in water extracts of thawing forest soils. Significant levels for the interactive effects of vegetation type (V) and soil WFPS (W) on each index are shown inside each sub-plot. Error bars reflect the standard error of Fmax and contribution of three components in soil water extracts between replicates, respectively (*n* = 3 for all treatments).

Fmax and their contributions of the three components were all significantly affected by the WFPS alone (*p* < 0.001) and interactively by vegetation type and WFPS (*p* < 0.05) (Fig. 3). Fmax of component 1 was significantly different in the two forest soil extracts, especially at 90% WFPS (*p* = 0.001) (Fig. 3a). Fmax of the three components in water extracts of the two forest soils varied little with low values from 10% to 30% WFPS and it generally increased with increasing WFPS with a range from 30% to 70% WFPS (Fig. 3a–c). Except for the Fmax of component 1 in the BKPF soil extracts, Fmax of the three components in the water extracts of the two forest soils showed a decrease tendency from 80% to 90% WFPS, which was significant for the Fmax of the three components in the WBF soil extracts (*p* < 0.05) (Fig. 3a–c).

From 10% to 30% WFPS, the component 1 contributed 43% to 78% and component 2 contributed 6% to 37% of the three components (Fig. 3d–f). Within a range from 40% to 90% WFPS, the component 2 contributed mostly (48% to 90%) of the three components, and both components 1 and 3 had a smaller contribution (2% to 33%) (Fig. 3d–f). Vegetation type and WFPS alone and interactively affected the contribution of the component 3 (*p* < 0.05), which ranged from 8% to 20% of the three components (Fig. 3f).

### CO_2_ flux, microbial biomass and spectral properties of DOM

**Figure 4:**
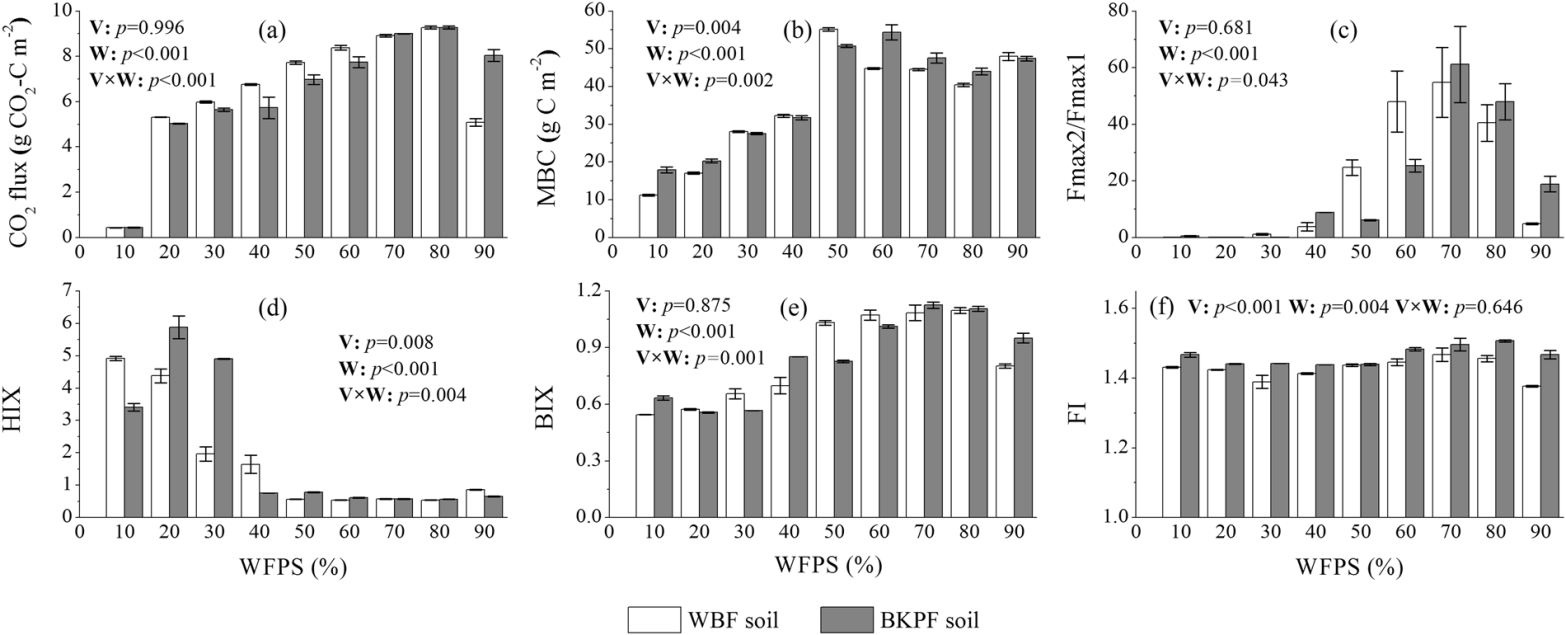
Effects of soil moisture on soil CO_2_ flux, microbial biomass and spectral properties of DOM released into the soils during thawing. Significant levels for the interactive effects of vegetation type (V) and soil WFPS (W) on each index are shown inside each sub-plot. Error bars reflect the standard error of soil CO_2_ flux, microbial biomass and spectral properties of DOM between replicates, respectively (*n* = 3 for all treatments).

The effects of soil moisture on CO_2_ flux, microbial biomass and spectral properties of DOM released into the soils during thaw were shown in Fig. 4. CO_2_ fluxes from WBF and BKPF soils during thaw were extremely low at 10% WFPS and then gradually increased from 20% to 80% WFPS; they decreased from 80% to 90% WFPS, especially for those from the WBF soil (*p* < 0.001). Microbial biomass carbon (MBC) in the WBF and BKPF soils increased from 10% to 50% WFPS, reached a maximum at 50–60% WFPS and then varied little till 90% WFPS. Soil WFPS and in combination with vegetation type significantly affected the soil MBC and CO_2_ fluxes from the two forest soils during thaw (*p* ≤ 0.002). The ratio between Fmax of component 2 and Fmax of component 1 (Fmax2/Fmax1) for DOM in water extracts of the two forest soils remained to be extremely low values from 10% to 30% WFPS, gradually increased from 30% to 70% WFPS and then decreased from 70% to 90% WFPS. Humification index (HIX) of DOM in water extracts of WBF soil gradually decreased from 10% to 50% WFPS, whereas for water extracts of BKPF soil, it remained to be above 3.0 from 10% to 30% WFPS and sharply dropped below 1.0 at 40% WFPS. From 50% to 90% WFPS, HIX of DOM in water extracts of the two forest soils always remained to be below 1.0 with little variations. Vegetation type and soil moisture significantly influenced the HIX of DOM in the soil water extracts singly and interactively (*p* < 0.01). Biological index (BIX) of DOM in water extracts of the two forest soils remained to be constant from 10% to 30% WFPS, and it gradually increased from 40% to 70% WFPS. Fluorescence index (FI) of DOM in water extracts of the two forest soils ranged from 1.38 to 1.51, and it was significantly affected by soil WFPS and vegetation type alone (*p* ≤ 0.004). Similar to the soil CO_2_ flux and Fmax2/Fmax1 ratio, both BIX and FI of DOM in the soil water extracts decreased from 80% to 90% WFPS, respectively, especially for those in the WBF soil (*p* ≤ 0.01).

### Relationships among CO_2_ flux, microbial biomass, biodegradation, components and spectral characteristics of DOM

**Figure 5:**
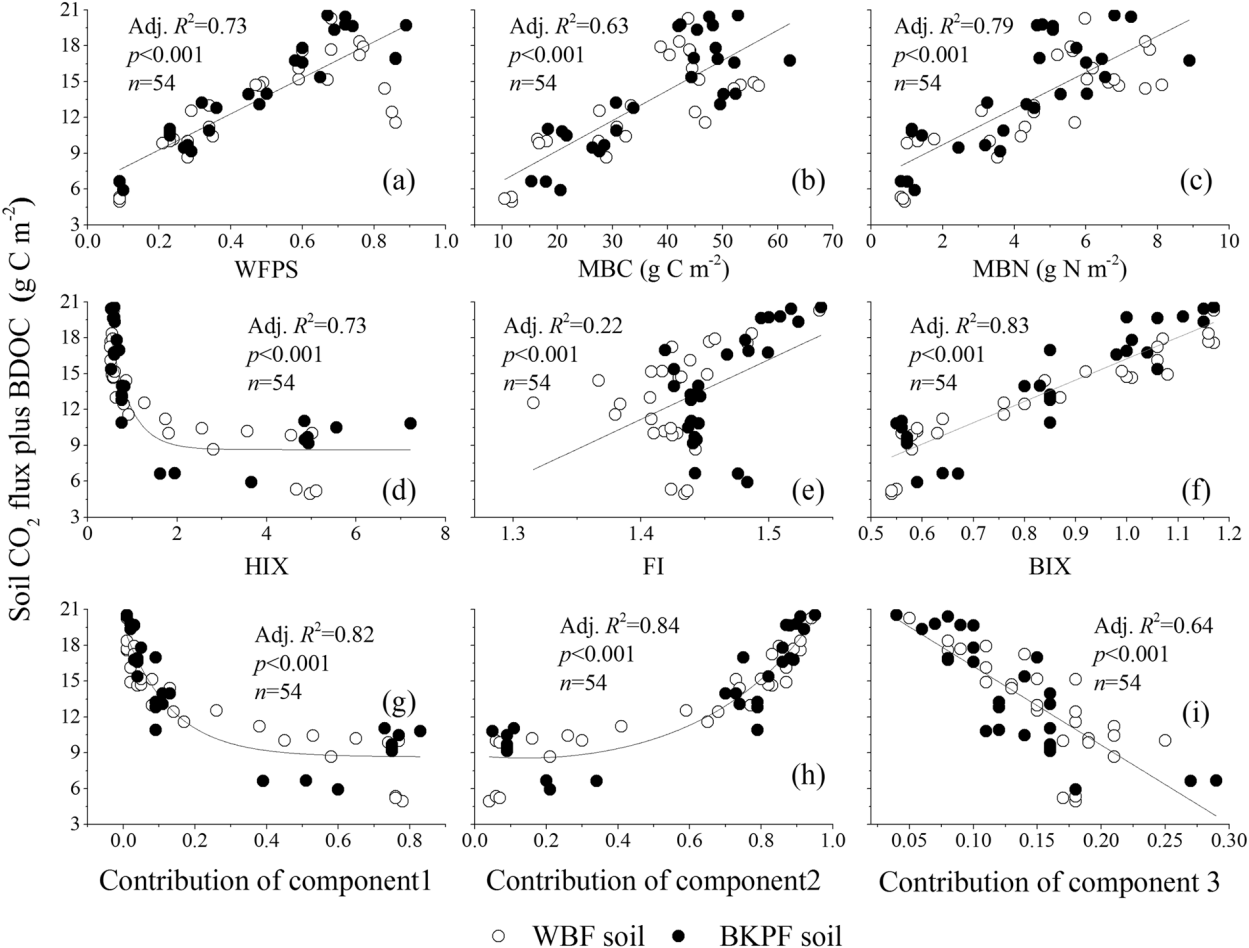
Relationships of the soil potential organic carbon decomposition (CO_2_ flux plus BDOC) against the soil WFPS, microbial biomass, the contribution of three components and spectral properties of DOM.

The Fmax of component 1 in water extracts of the two forest soils during thaw was positively correlated to the NBDOC, NBDON and SUVA_254_, respectively (*p* < 0.001) (Supplementary Figure S2a–c and Table S2). The Hix was positively correlated to the contribution of component 1 (*p* < 0.001) but negatively correlated to the contribution of component 2 and the Fmax2/Fmax1 ratio, respectively (*p* < 0.001) (Supplementary Figure S2d–f and Table S2). The Fmax of component 3 in water extracts of the two forest soils during thaw was positively correlated to the MBC, MBN and BIX, respectively (*p* < 0.001) (Supplementary Figure S2g–i and Table S2). NBDOC and NBDON concentrations in water extracts of the two forest soils during thaw were both positively correlated to the SUVA_254_, respectively (*p* < 0.001) (Supplementary Figure S3 and Table S2). The CO_2_ flux plus BDOC concentration was positively correlated to the WFPS, MBC, MBN, FI, BIX and the contribution of component 2 (*p* < 0.001), respectively and negatively correlated to the HIX and the contribution of components 1 and 3 (*p* < 0.001), respectively (Fig. 5).

## Discussion

DOM derived from different terrestrial ecosystems has qualitatively similar fluorescence components, due to the presence of humic acid-like substances and protein-containing aromatic amino acids^29^. The location and outline of component 1 in this study was the same as what reported by *Huang et al*.^30^ (λ_Ex_ = 250 nm, λ_Em_ = 434 nm) and defined as humic acid-like component^22^. A significant and positive relationship of Fmax of component 1 against the NBDOC, NBDON and SUVA_254_ (Supplementary Figure S2a–c) suggested that this component was more difficult to be biodegraded than the other two components. This was supported by the fact that the contribution of component 1 to all the three components was positively correlated to the HIX (Supplementary Figure S2d) and negatively to the soil potential organic carbon decomposition (Fig. 5g). This component was also reported by *Nishimura et al*.^31^ (λ_Ex_ = 260 nm, λ_Em_ = 460 nm) in DOM leached from fresh fallen litter and senescent litter during the early decomposition. The location and outline of component 2 in this study was similar to those of component 3 reported by *Ohno et al*.^32^ (λ_Ex_ < 240 nm, λ_Em_ = 465 nm) and component 5 reported by *Hunt and Ohno* ^29^ (λ_Ex_ < 240 nm, λ_Em_ = 340 nm), which was classfied as fulvic acid-like component^22, 33^. The contribution of component 2 was negatively correlated to the HIX (Supplementary Figure S2e) and positively correlated to the soil potential organic carbon decomposition (Fig. 5h), which indicated that component 2 was relatively easier to be biodegraded than the other two components. According to previous studies^22, 24, 34^, the component 3 in this study was protein-like component (e.g. tyrosine and tryptophan), which originates from microbial biomass. This was well documented by the positive relationship of the Fmax of component 3 against the MBC, MBN and BIX (Supplementary Figure S2g–i). The contribution of component 3 was negatively correlated to the soil potential organic carbon decomposition (Fig. 5i), which was probably attributed to its quick mineralization during thaw. This protein-like component was hard to be observed in soil extracts^23, 32^ because of its rapid biodegradation within few minutes to several hours^35^. *Guigue et al*.^36^ reported a same protein-like component in soil extracts as in this study, which varied significantly with vegetation types (Fig. 3f). As shown in Fig. 3, soil moisture could significantly affect the Fmax of three components and their contributions, and this effect could significantly vary with vegetation types.

**Figure 6:**
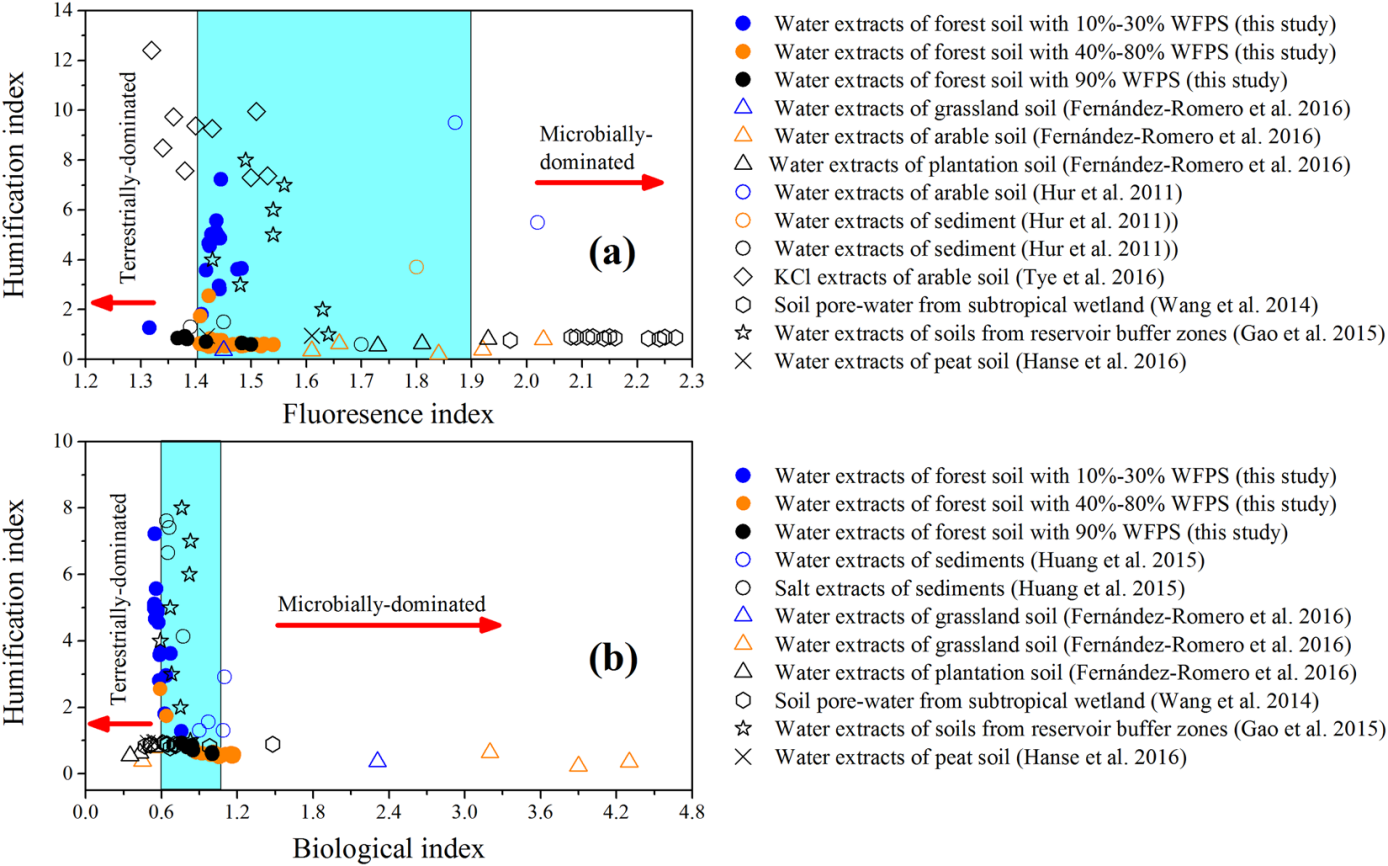
Comparison plots of humification index versus (**a**) fluorescence index and (**b**) biological index for water extracts of forest soils during thaw, together with different DOM data refering to results from Fernández-Romero *et al*.^62^, Hur *et al*.^63^, Tye *et al*.^64^, Wang *et al*.^65^, Gao *et al*.^66^, Hansen *et al*.^46^, and Huang *et al*.^67^. The shaded regions represent a mixed source of DOM.

Low soil moisture (e.g. 10% WFPS) via air-drying process prior to freezing benefited DOM accumulation in forest soils during thaw (Fig. 1) but not for the recovery of soil microorganisms after frost (Fig. 4b). This DOM accumulation, especially BDOM accumulation probably resulted from the amount of DOM released during air-drying process^37^ and the relative low microbial activity during thaw (Fig. 4a). In the present study, at less than 30% WFPS, DOM in water extracts of forest soils during thaw was dominated by the component 1 (Fig. 3d) with high HIX (Fig. 4d) and low BIX values (Fig. 4e), which indicated that the DOM was mainly composed of complex molecules with high molecular weight and biological origin contributed little to the DOM^38, 39^. Together with previous studies, it was inferred that during thaw, the biological origin of DOM in forest soils became minor at less than 30% WFPS (Fig. 6b). Previous experiments without freezing treatment showed that air-drying process could increase the amount of DOM released into the soil and the DOM was mainly consisted of humic acid-like materials^37^ and high molecular weight compounds^40^. Very few studies^10, 41^ reported the components or stability of DOM in soil after freezing treatment with low water content. *Haei et al*.^10^ reported that SUVA_254_ values in water extracts of forest soil (30% water holding capacity) after freezing at −12 °C and −6 °C were 0.04 and 0.07, respectively, which is larger than that (0.03) in water extracts of soil with 30% WFPS in this study. Laboratory experiment conducted by *Chen et al*.^41^ showed that, after freezing at −25 °C for 7d, water extracts of arable soil at 20% WFPS had a significant higher DOC concentration and lower SUVA_280_ value compared to the soil at 80% WFPS. Hence, the amount and bioavailability of DOM released into the soil after frost under low moisture conditions was dependent on soil types, the degree of soil drying and freezing conditions.

High antecedent soil moisture prior to freezing can enhance the intensity of ice formation, which increases physical damages to soil aggregates and microorganisms and thus can accelerate the release of DOM during soil thaw^4, 10^. This could to some extent explain an increasing concentration of DOM released into the forest soils after frost from 30% to 90% WFPS (Fig. 1). With an increase of soil moisture from 30% to 90% WFPS, the concentrations of BDOC and BDON in the forest soils after frost generally increased, with a maximum of BDOC at 90% WFPS and BDON at 70% WFPS, respectively (Fig. 1). Probably, there was a different accumulation of BDOC and BDON released into the forest soils after frost under high soil moisture conditions. The increase in BDOM concentration of forest soils after frost under high soil moisture conditions was partly attributed to the release of fulvic acid-like component into the soils (Fig. 3e), which was mainly composed by the easily biodegradable materials with low molecular weight. In the moist tundra of the arctic Alaska foothills, DOM in flowing free water of soil was also dominated by fulvic acid-like component during the early stage of spring thaw^41^. And, by simulating spring thaw in the laboratory, *Michaelson et al*.^42^ reported that hydrophilic neutral fraction accounted for 71% of the DOC in water leachates from intact thawing soil core. Field experiment conducted by *Cannavo et al*.^43^ showed that each heavy rainfall (60 mm) could bring a sharp decrease of HIX for soil water extracts from 2.6 to 1.3. In the present study, by wetting soil from 30% to 40% WFPS, the HIX value of forest soil water extracts during thaw decreased from 2.0–4.9 to 0.8–1.6, especially for the BKPF soil, and it remained to be below 1.0 with increasing soil moisture (Fig. 4d). Laboratory experiment conducted by *Haei et al*.^10^ showed that, after different numbers of freeze-thaw cycles with three levels of soil moisture (30%, 60% and 90% WFPS), SUVA_254_ values in water extracts of forest soils increased with increasing soil moisture and this effect was more pronounced under the conditions of lower freezing temperature and fewer freeze-thaw cycles. Forest soils with water content close to field capacity were incubated by *Schmitt et al*.^44^ and they reported that, compared to the soil incubated at 5 °C constantly, DOM in the soil experienced three freeze-thaw cycles tended to be more humified with more lignin component but had less total sugar component. And this humification degree could be increased by a decrease of freezing temperature from −3 °C to −13 °C^44, 45^. According to the results of this present study and the previous studies, it can thus be reasonably concluded that increasing antecedent soil moisture prior to freezing can increase the amount and bioavailability of DOM released into the soil during thaw, and this effect can depend on vegetation types and freeze-thaw conditions.

With increasing soil moisture from 40% to 80% WFPS, the FI values of soil water extracts varied little (1.38–1.51), but the increasing BIX, soil microbial biomass and microbial respiration (Fig. 4) revealed an increasing role of microbial functions in the DOM turnover^39, 46^. As shown in Fig. 6, the origin of soil microorganisms to the DOM became greater under high soil moisture conditions. The BIX was positively correlated to soil moisture (Supplementary Table S2) and this relationship was also reported by *Wang et al*.^47^, who indicated that BIX was a valuable proxy for distinguishing DOC sources in different environments. Furthermore, the same trend of the variations in BIX and CO_2_ flux towards increasing soil WFPS (except for 10%) (Fig. 4a,e) and the significant positive relationship between BIX and the soil potential organic carbon decomposition (Fig. 5f) indicated that BIX could be an ideal index to reasonably reflect DOM biodegradation or mineralization in the soils during thaw. Hence, BIX and in combination with FI and HIX could reflect the sources and stability of DOM in different soils during thaw (Fig. 6). And in this study, HIX and BIX further reflected properties of the three components in DOM (Supplementary Figure S2). It is an innovation to study the components and stability of DOM in soils during thaw by using EEM spectrophotometry combined with PARAFAC and in the same time, measuring soil C mineralization and DOM biodegradation. Future research is needed to focus on the effects of different freezing-thaw conditions (e.g., freezing temperature, freeze-thaw frequency and thaw temperature) on the quantities, compositions and stability of DOM in different soils during thaw and their related microbial mechanisms.

In the present study, the SUVA_254_ was positively correlated to soil WFPS (Supplementary Table S2), which is consistent with *Haei et al*.^10^, who reported that increasing soil moisture prior to freezing (−6 °C) increased SUVA_254_ in water extracts of thawing soil. Under the experimental conditions, the SUVA_254_ was positively correlated to the NBDOM concentration and CO_2_ flux but not significantly correlated to the BDOC concentration (Supplementary Figure S3 and Table S2). The HIX was negatively correlated to the BDOC, CO_2_ flux and the soil potential organic carbon decomposition during thawing period (Fig. 5 and Supplementary Table S2). Hence, compared to the SUVA_254_, HIX reflected the biodegradation or mineralization of soil DOM better. This is consistent with *Marschner and Bredow* ^20^, who reported that the SUVA_254_ did not correlate well with DOM biodegradability because nonaromatic compounds of DOM may vary significantly in biodegradability which depended upon their degree of polymerization or oxidation^29^. The relationship between HIX and SUVA_254_ still remained controversial. A significant positive correlation between the two parameters was respectively reported by *Embacher et al*.^48^ in water extracts of arable soils (*r* = 0.91) and by *Williams et al*.^49^ in watershed from mixed land use in Canada (*r* = 0.74). However, the present study and *Zhang et al*.^21^ reported a significant negative relationship between the two parameters (*r* < −0.73). With high soil moisture (50% to 90% WFPS) prior to freezing, DOM released into the forest soils during thaw was dominated (69% to 90%) by the fluvic acid-like component (Fig. 3e), and high SUVA_254_ but low HIX values of the DOM reflected that this component was less condensed but ring-structured with high mobility^21^. The mobility of DOM is supported by the results of field experiments conducted by *Michaelson et al*.^42^, who reported that DOM fraction was similar in flowing free water of soil and water from adjacent stream during the early stage of spring thaw and dominated by the fulvic acid fraction. In conclusion, after freezing with high soil moisture during winter (>50% WFPS), forest soils would release relatively large amounts of fulvic acid-like DOM with high aromaticity but low humification degree, which is easily to be biodegraded and emitted as CO_2_ or lost with horizontal sub-surface flow during spring snow thaw.

## Methods

### Site description and collection of forest soil

The study area is located near the National Research Station of Changbai Mountain Forestry Ecosystem (42°24′N, 128°6′E) in Jilin province, northeastern China (Supplementary Figure S4) with continental temperate climate. Based on regular meteorological measurements of the station during the period from 2004 to 2012, continuous surface soil freezing (daily maximum temperatures below 0 °C) lasted for 52 d to 89 d each year, and the average air temperature and average soil temperature at 10 cm during this period ranged from −12.6 °C to −17.7 °C and from −2.0 °C to −8.1 °C, respectively. Soil profile in winter can be frozen down to 1.0–1.5 m depth and a complete disappearance of such frozen soil layer normally occurs in the middle of May each year^4^. The mature broadleaf and Korean pine mixed forest (BKPF) and an adjacent white birch forest (WBF) were selected for soil sampling. Compared to the BKPF stand, snow under WBF stand melts more quickly during spring thaw period due to its relatively lower canopy density and phototaxis. Due to the relatively higher soil permeability, soil moisture under WBF stand is generally lower compared to BKPF soil. Besides, the fluorescence components^22^ of soil water-soluble DOM under the two forest stands were different (Supplementary Figure S5). Soil samples (0–10 cm) were collected in October 2014 using an 8-cm diameter auger separately under the two forest floors after removing the ground surface mulch. To collect composite soil samples, eighteen 1 m × 1 m plots were selected in each forest stand. All soil samples were kept separately in air-tight plastic bags and rapidly transported to the laboratory within 24 h. Soil samples from each forest stand were mixed thoroughly, sieved (<2-mm) to remove small stones and debris, and then stored in the dark at 4 °C prior to incubation and analysis of soil properties. The dark brown forest soil under the two forest stands is originated from volcanic ash. The particle size distribution of surface forest soil (0–10 cm) was determined using a laser diffractometer (Mastersizer 2000, Malvern, UK), and the volume percentage of sand (50–2000 μm), silt (2–50 μm) and clay (<2 μm) was shown in Supplementary Table S1.

### Measurement of soil C and N pools

Triplicate soils were dried at 105 °C for 24 h to determine moisture content. Fresh soil pH (soil/water, 1/2.5, *w*/*w*) was measured with a portable pH meter (PB-10, Sartorius, Germany). Total C and N concentrations in soil samples were measured using an elemental analyzer (FLASH 2000, Thermo Scientific, USA). Fresh forest soils (5.0 g) were extracted by shaking with 25 mL of deionized water for 30 min on an end-over-end shaker. The suspensions were centrifuged at 6400 *g* for 5 min and then filtered into 50-mL plastic bottles via cellulose-acetate membrane filters (0.45 μm pore size)^50^. Concentrations of NH_4_^+^-N, NO_3_^−^-N plus NO_2_^−^-N, total N (TN), and dissolved organic C (DOC) in the soil water extracts were measured using a continuous flow analyzer (SAN^++^, SKALAR, the Netherlands) and calibrated using 7 levels of certified solution standards. The correlation coefficients of calibration curves were ensured to be above 0.999 and, every 10 soil extract samples, one sample with known concentration was normally measured on-line to correct the instrumental bias. Concentrations of soil dissolved organic N (DON) were calculated according to the differences between TN and inorganic N (NH_4_^+^-N and NO_3_^−^-N plus NO_2_^−^-N) concentrations in soil water extracts. Concentrations of soil microbial biomass C (MBC) and N (MBN) were measured by the chloroform fumigation and extraction method and calculated by the differences of K_2_SO_4_-extractable DOC and TN pools between fumigated and non-fumigated soils and divided by 0.45^51–53^, assuming that fumigation causes a release of microbial N in the same proportion as for microbial C. TN and DOC in the soil K_2_SO_4_ extracts were measured using a continuous flow analyzer as above. Main properties of forest soils under WBF and BKPF stands were shown in Supplementary Table S1.

### Layout of freezing-thawing incubation experiment

Packed soil cores were made in accordance with bulk densities of BKPF and WBF soils in the field^54^. Homogenized initial fresh soils (63.2 g of dry weight) were transferred into PVC cylinders (3.7 cm in diameter and 12 cm in height) as a soil core with 27% WFPS. Amount of deionized water was sprayed onto the homogenized soil before packing to reach WFPS levels of 30%, 40%, 50%, 60%, 70%, 80% and 90%. Another six soil cores were air-dried to 10% WFPS in advance, and then three of them were wetted with deionized water to 20% WFPS. Every treatment was replicated three times, giving a total of 54 packed soil cores (9 moisture levels × 2 vegetation types × 3 replicates). After wetting, all the soil cores were immediately frozen at −8 °C in the freezer. The duration and temperature of freezing was simulated according to winter frost from late December to next February near the study area^4^. After freezing for 60 days, all the soil cores were immediately sealed inside gas tight PVC cylinders (760 mL) with a gas sampling port equipped with 3-way stopcock separately. In accordance with maximum air temperature of the study area in late spring when soil freezing-thawing cycles intensively occur in the field, the soil cores were incubated at 10 °C in an incubator (LRH-250, Yiheng Instruments, Shanghai) without light for 10 days to simulate the soil thawing process. Deionized water was added for each soil core by weighting every three days during the 10-day incubation to avoid evaporation. For measurement of DOC stability as indicated by C loss, CO_2_ fluxes were measured immediately following thawing of frozen soils. Gas sampling was performed from each cylinder at 6, 12, 24, 36, 48, 72, 96, 120, 144, 168, 192, 216 and 240 h after the incubation was initiated. For each sampling, headspace gas samples of 30 ml were collected using 50-mL polypropylene syringes equipped with 3-way stopcock at 0 and 6 or 12 h after sealing the cylinders. Each time before gas sampling, all PVC cylinders were taken outdoors to be well ventilated for 10 min and then sealed until the next sampling time. The concentrations of CO_2_ in headspace gas samples were quantified by a gas chromatograph (Agilent 7890 A, Franklin, USA) equipped with a flame ionization detector (FID)^4^. The detector responses were calibrated using a certified gas standard, which contains 2.02 mL L^−1^ CO_2_ in air. Main properties of soil cores including moisture, bulk density, DON, DOC, MBC, and MBN were measured immediately when the last gas sampling finished, as mentioned above. Due to freezing effect, soil WFPS inside each core at the end of incubation was calculated by soil bulk density and moisture according to *Franzluebbers* ^55^. Water extracts of soil samples were stored in the dark at 4 °C for a week prior to both DOM biodegradation and excitation-emission matrix fluorescence analysis of DOM.

### Measurement of DOM biodegradation

Each water extract of soil (10 mL) with 0.1 mL of inoculum suspension was incubated in 60 mL tubes at 25 °C in the dark for 7 d when the specific ultraviolet absorbance at 254 nm of the extract was nearly stable according to our previous study^27^. The inoculum was prepared by shaking a mixture of 25 g of forest topsoil incubated at a water capacity of 60% for two weeks at 20 °C with 50 mL of 4 mmol L^−1^ CaCl_2_ solution for 30 min, followed by centrifugation at 6400 *g* for 10 min^26^. The tubes were shaken with a vortex mixer (QL-901, Qilinbeier Instruments, China) at 12 h intervals throughout the incubation period. Biodegradable DOC (BDOC) and DON (BDON) concentrations were calculated by differences of DOC and DON concentrations in each soil extract before and after the incubation. Non-biodegradable DOC (NBDOC) and DON (NBDON) concentrations were considered DOC and DON concentrations in each soil extract at the end of the incubation. The methods for measuring DOC and DON concentrations were mentioned above. The biodegradation of the inoculum C and N were determined from a control sample containing ultra-pure water and inoculum and subtracted from the soil extract samples.

### Fluorescence and ultraviolet analysis of DOM and PARAFAC analysis

EEM fluorescence of each soil water extract was measured using a fluorometer (Fluoromax-4, Horiba, USA). The emission scan was made from the wavelengths of 290 nm to 550 nm with 2 nm increments at a stepwise increase of 5 nm for the excitation wavelengths (λ_Ex_) from 240 nm to 455 nm. All fluorescence spectra were acquired after correction for variations in lamp intensity with respect to time using ratio mode (sample/reference). Inner filter correction^56^ was carried out using absorbance spectrum measured with a spectrophotometer (U-2000, Hitachi, Japan). After then, the EEM of Milli-Q water was subtracted from the sample EEM. Finally, each EEM was calibrated to the water Raman signal^57^ and then corrected to the DOC concentration of each soil extract, and was expressed in RU mg^−1^ C. The SUVA_254_ was calculated from the UV absorbance at 254 nm divided by the DOC concentration (mg C L^−1^) and the path length of the quartz cell of the spectrophotometer (cm), which is typically used for indicating the aromaticity of DOM^58^, and is expressed as L mg^−1^ C cm^−1^. Humification index (HIX) was calculated by the ratio of two integrated regions of emission scan (sum of 436 nm to 480 nm divided by the sum of 300 nm to 344 nm) with λ_Ex_ at 255 nm, indicating the relative humification of soil extracts^59^. Biological index (BIX) was calculated by the ratio of emission intensity at 380 nm to that at 430 nm with λ_Ex_ at 310 nm, indicating the relative contribution of autochthonous DOM in soil extracts^39^. Fluorescence index (FI) was calculated by the ratio of emission intensity at 470 nm to that at 520 nm with λ_Ex_ at 370 nm, providing a metric for distinguishing DOM derived from allochthonous or autochthonous sources^60^.

PARAFAC analysis was performed in MATLAB (R2012a) with the drEEM toolbox (version 1.0)^61^ with non-negativity constraint including EEMs of all the 54 water extracts in this study. One EEM with high leverage was removed as an outlier from the subsequent calculations. A model with three components was validated through split half analysis and explained 99.45% of the variation in the data. Fluorescence intensity at the maximum (Fmax) was used to reflect the quantity of each component, and Fmax/∑Fmax (the ratio of Fmax for each component to the sum of Fmax for all the three components) indicated the relative contribution of each component in the DOM.

### Calculation and statistical analysis

Cumulative CO_2_ fluxes during the 10-day incubation were calculated as the sum of CO_2_ flux for each sampling, and were expressed in g CO_2_-C m^−2^. Soil CO_2_ flux plus BDOC were calculated to represent the potential organic carbon decomposition during soil thaw, and were expressed in g C m^−2^. Means and standard errors of three replicates were calculated. For each treatment, EEMs from three replicates were averaged and then plotted using SigmaPlot (version 12.0, Systat Software, Inc., USA). All measured variables were examined for normality (Shapiro-Wilk test) and homogeneity (Levene’s test) of variance and transformed where necessary. One-way analysis of variance (ANOVA) was used to assess the effects of soil moisture on the concentrations of BDOC, NBDOC, BDON and NBDON. A two-factor repeated ANOVA was used with vegetation type and soil moisture to assess their interactive effects on the CO_2_ flux, MBC, HIX, BIX, FI, Fmax and the contribution of each component, and the ratio between Fmax of component 2 and component 1 (Fmax2/Fmax1). The relationships among the biodegradation and spectral properties of DOM, CO_2_ flux and Fmax and the contribution of each component were shown in scatter plots and fitted with linear or nonlinear regressions. Significant pairwise differences were determined at the *p* < 0.05 level using student *T*-test. All statistical analyses were conducted with the software SPSS for Windows (version 19.0, IBM Corp., USA).

## Electronic supplementary material

Supplementary information
