# Supplementary material for: Antecedent soil moisture prior to freezing can affect quantity, composition and stability of soil dissolved organic matter during thaw
Source: Sci Rep. 2017 Jul 25;7:6380. doi: 10.1038/s41598-017-06563-8 (PMC5526942; doi:10.1038/s41598-017-06563-8)

Supplementary Information

Antecedent soil moisture prior to freezing can affect quantity, composition and stability of soil dissolved organic matter during thaw

Haohao Wu[1,2], Xingkai Xu[1,2,*], Weiguo Cheng[3], Pingqing Fu[1], and Fayun Li[4,5]

[1] State Key Laboratory of Atmospheric Boundary Layer Physics and Atmospheric Chemistry, Institute of Atmospheric Physics, Chinese Academy of Sciences, Beijing 100029, China.

[2] Department of Atmospheric Chemistry and Environmental Science, College of Earth Science, University of Chinese Academy of Sciences, Beijing 100049, China.

[3] Faculty of Agriculture, Yamagata University, Tsuruoka 997-8555, Japan.

[4] Institute of Eco-Environmental Sciences, Liaoning Shihua University, Fushun 113001, China.

[5] National & Local United Engineering Laboratory of Petroleum Chemical Process Operation, Optimization and Energy Conservation Technology, Liaoning Shihua University, Fushun 113001, China.

Corresponding Author:[*] Tel: +86-10-82085510; fax: +86-10-62041393;

email: xingkai_xu@mail.iap.ac.cn

The supplementary information has 8 pages including two tables and five figures.

Table S1 Main properties of forest soils under WBF and BKPF stands.

Vegetation type	Moisture (%, *w*/*w*)	Sand	Silt (%, *v*/*v*)	Clay	pH (water)	Total C (mg C g^{-1})	Total N (mg N g^{-1})	NO_3^--N (μg N g^{-1})	NH_4^+-N (μg N g^{-1})	DON (μg N g^{-1})	DOC (μg C g^{-1})	MBN (mg N kg^{-1})	MBC (mg C kg^{-1})	MBC:MBN ratio
WBF	32.5	24.1	74.8	1.1	5.52	56.8	4.5	23.2	3.5	6.1	81.5	238	1415	5.9
BKPF	32.3	31.9	67.2	0.9	5.58	80.4	7.1	30.4	3.3	6.8	89.6	193	1202	6.2

Table S2 Correlation coefficients among soil CO_2 flux, microbial biomass, concentrations and biodegradation as well as spectral properties of DOM.

	DOC	BDOC	NBDOC	DON	BDON	NBDON	CO_2	MBC	MBN	MBC:MBN	WFPS	a_{254}	$SUVA_{254}$	Fmax1	Fmax2	Fmax3	Comp1C	Comp2C	Comp3C	Fmax2/Fmax1	FI	HIX	BIX
DOC	1																						
BDOC	0.81**	1																					
NBDOC	0.92**	0.56**	1																				
DON	0.95**	0.74**	0.92**	1																			
BDON	0.65**	0.78**	0.51**	0.63**	1																		
NBDON	0.67**	0.29*	0.80**	0.74**	0.09	1																	
CO_2	0.48**	0.07	0.68**	0.56**	0.08	0.64**	1																
MBC	0.54**	0.20	0.68**	0.61**	0.12	0.68**	0.61**	1															
MBN	0.53**	0.15	0.71**	0.64**	0.16	0.70**	0.74**	0.89**	1														
MBC:MBN	-0.21	0.13	-0.40**	-0.32*	0.02	-0.51**	-0.65**	-0.53**	-0.81**	1													
WFPS	0.67**	0.31*	0.81**	0.75**	0.17	0.85**	0.74**	0.74**	0.77**	-0.58**	1												
a_{254}	0.85**	0.51**	0.91**	0.86**	0.48**	0.74**	0.79**	0.65**	0.73**	-0.50**	0.73**	1											
$SUVA_{254}$	0.68**	0.28	0.79**	0.73**	0.25	0.73**	0.88**	0.64**	0.79**	-0.64**	0.77**	0.92**	1										
Fmax1	0.67**	0.26	0.81**	0.69**	0.15	0.79**	0.76**	0.72**	0.74**	-0.53**	0.83**	0.87**	0.90**	1									
Fmax2	0.76**	0.33*	0.89**	0.79**	0.30*	0.79**	0.85**	0.72**	0.80**	-0.62**	0.80**	0.96**	0.94**	0.89**	1								
Fmax3	0.73**	0.32*	0.85**	0.77**	0.27*	0.78**	0.85**	0.70**	0.80**	-0.64**	0.80**	0.95**	0.95**	0.89**	0.99**	1							
Comp1C	-0.73**	-0.33*	-0.87**	-0.77**	-0.31*	-0.77**	-0.85**	-0.70**	-0.81**	0.65**	-0.76**	-0.95**	-0.93**	-0.83**	-0.98**	-0.98**	1						
Comp2C	0.77**	0.35*	0.89**	0.79**	0.32*	0.78**	0.83**	0.72**	0.80**	-0.62**	0.77**	0.96**	0.92**	0.87**	0.99**	0.97**	-0.98**	1					
Comp3C	-0.68**	-0.31*	-0.83**	-0.69**	-0.36**	-0.67**	-0.67**	-0.55**	-0.63**	0.42**	-0.61**	-0.84**	-0.79**	-0.78**	-0.82**	-0.77**	0.78**	-0.83**	1				
Fmax2/Fmax1	0.73**	0.32*	0.87**	0.77**	0.31*	0.76**	0.85**	0.701**	0.81**	-0.65**	0.76**	0.95**	0.93**	0.83**	0.98**	0.98**	-0.99**	0.99**	-0.79**	1			
FI	0.60**	0.42**	0.54**	0.50**	0.43**	0.25	0.36**	0.21	0.22	-0.02	0.20	0.61**	0.45**	0.51**	0.55**	0.52**	-0.49**	0.55**	-0.68**	0.51**	1		
HIX	-0.63**	-0.27	-0.77**	-0.69**	-0.21	-0.75**	-0.85**	-0.67**	-0.81**	0.68**	-0.73**	-0.87**	-0.90**	-0.75**	-0.91**	-0.92**	0.95**	-0.91**	0.66**	-0.94**	-0.31*	1	
BIX	0.72**	0.31*	0.85**	0.77**	0.31*	0.76**	0.86**	0.69**	0.81**	-0.64**	0.76**	0.94**	0.93**	0.83**	0.98**	0.98**	-0.99**	0.98**	-0.78**	0.99**	0.49**	-0.95**	1

Fmax1, Fmax2 and Fmax3 represent Fmax of component 1, 2 and 3, respectively. Comp1C, Comp2C and Comp3C represent the contribution of component 1, 2 and 3, respectively. *, p<0.05; **, p<0.01.

Figure S1 EEM fluorescence spectra of water extract of BKPF soil at 10% WFPS during thaw.

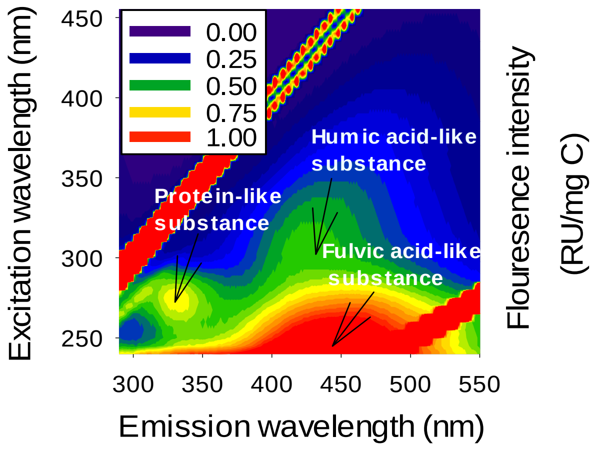

Figure S2 Relationships among spectral properties of DOM, NBDOM concentrations and soil microbial biomass during thaw.

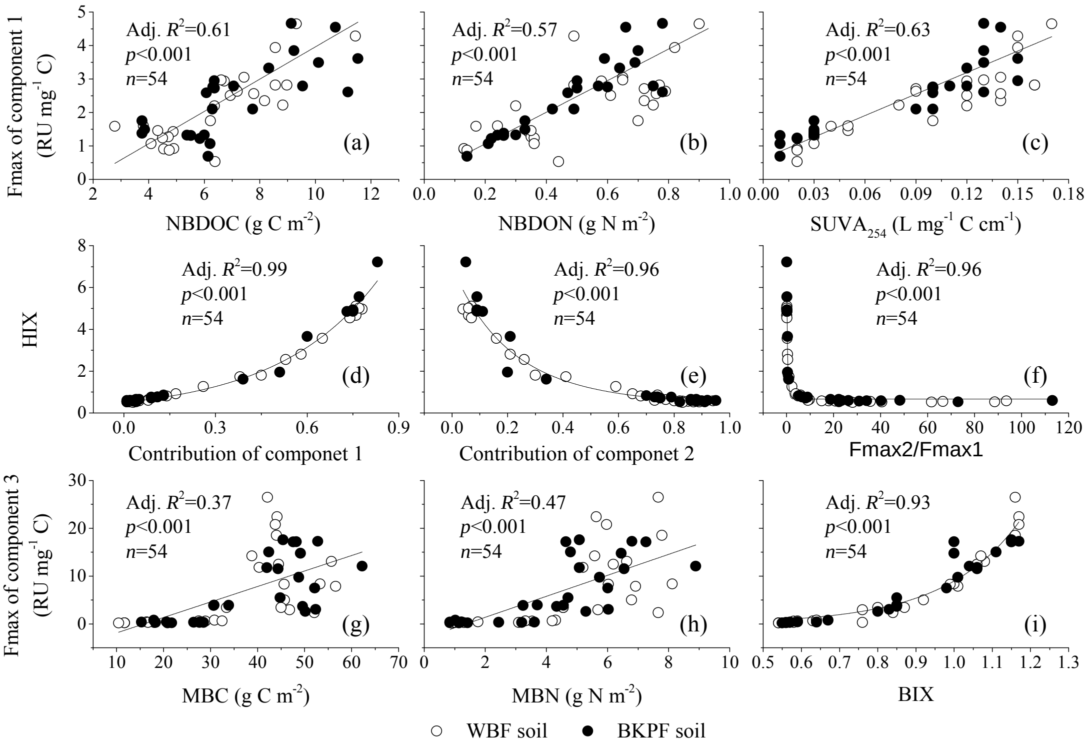

Figure S3 Relationships of $SUVA_{254}$ values against the NBDOC and NBDON concentrations in forest soils during thaw.

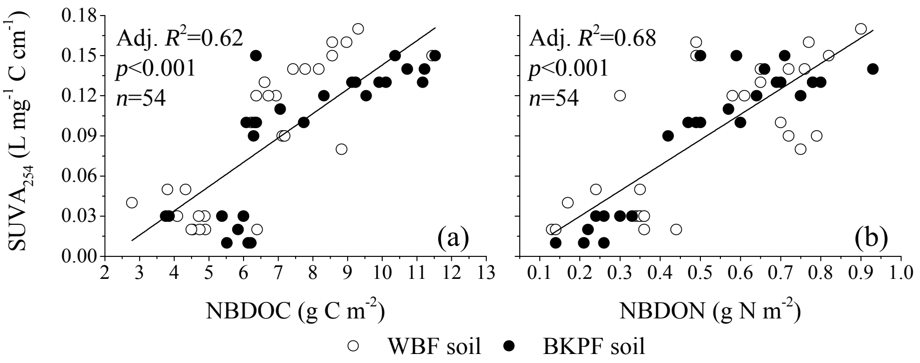

Figure S4 Map of the study area located near the National Research Station of Changbai Mountain Forestry Ecosystem (CBM station) in Jilin province, northeastern China. BKPF and WBF represent the mature broadleaf and Korean pine mixed forest and secondary white birch forest, respectively. Administrative boundaries of China were downloaded from the Global Change Research Data Publishing & Repository (http:// http://www.geodoi.ac.cn/). The figure was produced using ArcGIS 10.2 (http://www.esri.com/). The photographs were taken by H.H. Wu.

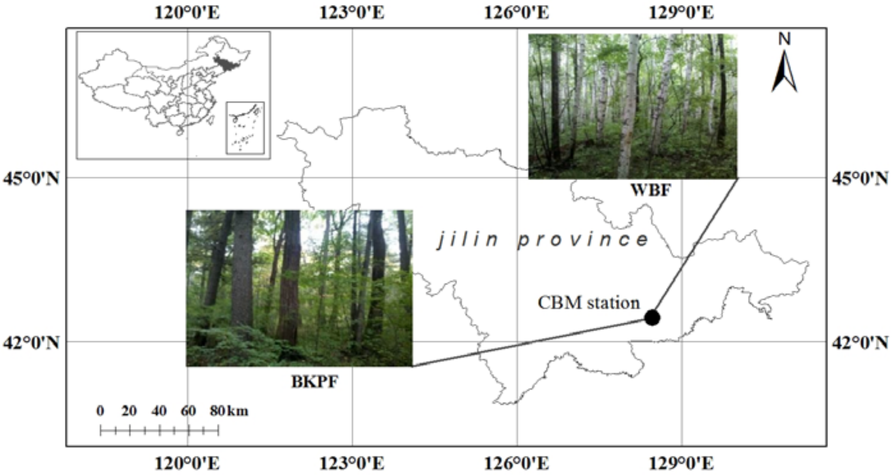

Figure S5 EEM fluorescence spectra of soil water extracts under WBF (a) and BKPF (b) stands.

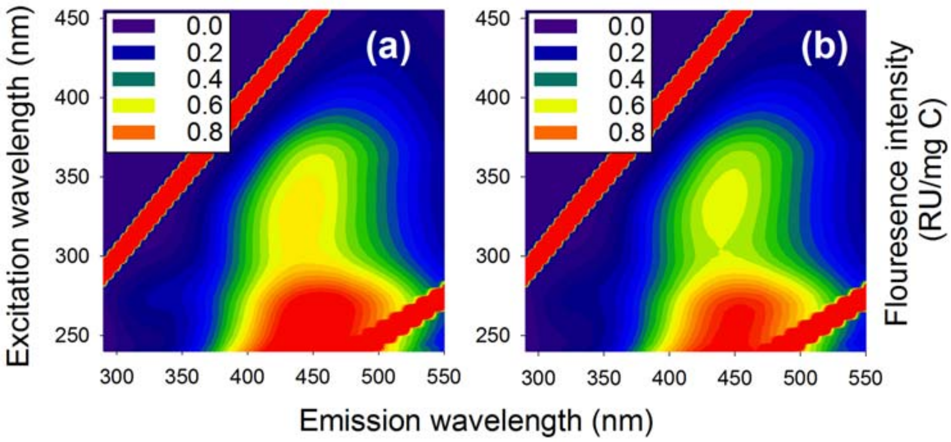

Supplement: Supplementary file 1 — Supplementary information [file 41598_2017_6563_MOESM1_ESM.pdf]
